# Supplementary material for: Lessons We Have Learned Regarding Seroprevalence in High and Low SARS-CoV-2 Contexts in Greece before the Omicron Pandemic Wave
Source: Int J Environ Res Public Health. 2022 May 17;19(10):6110. doi: 10.3390/ijerph19106110 (PMC9140933; doi:10.3390/ijerph19106110)
Supplement: Supplementary file 1 [file ijerph-19-06110-s001.zip › ijerph-1672354-supplementary.pdf]

Table S1. Number of SARS-COV-2 infections, hospitalizations and nonhospitalized cases by date in Malesina.

Weekly Update from the Civil Protection of the Municipality of Lokroi			
**Date**	**Active SARS-COV-2 Cases**	**Hospitalizations**	**Nonhopitalized Cases**
2021/1/4	3	1	2
2021/1/11	2	1	1
2021/1/18	5	1	4
2021/1/25	2	1	1
2021/2/1	12	1	11
2021/2/8	11	1	10
2021/2/15	17	4	13
2021/2/22	20	4	16
2021/3/1	186	11	175
2021/3/8	241	50	191
2021/3/15	164	64	100
2021/3/22	107	56	51
2021/3/29	74	42	32
2021/4/5	57	32	25
2021/4/12	45	13	32
2021/4/19	18	6	12
2021/4/26	13	2	11
2021/5/3	7	2	5
2021/5/10	14	2	12
2021/5/17	24	1	23
2021/5/24	16	3	13
2021/5/31	21	3	18
Sum	1059	301	758

Note: Provided by the the Civil Protection of the Municipality of Lokroi.

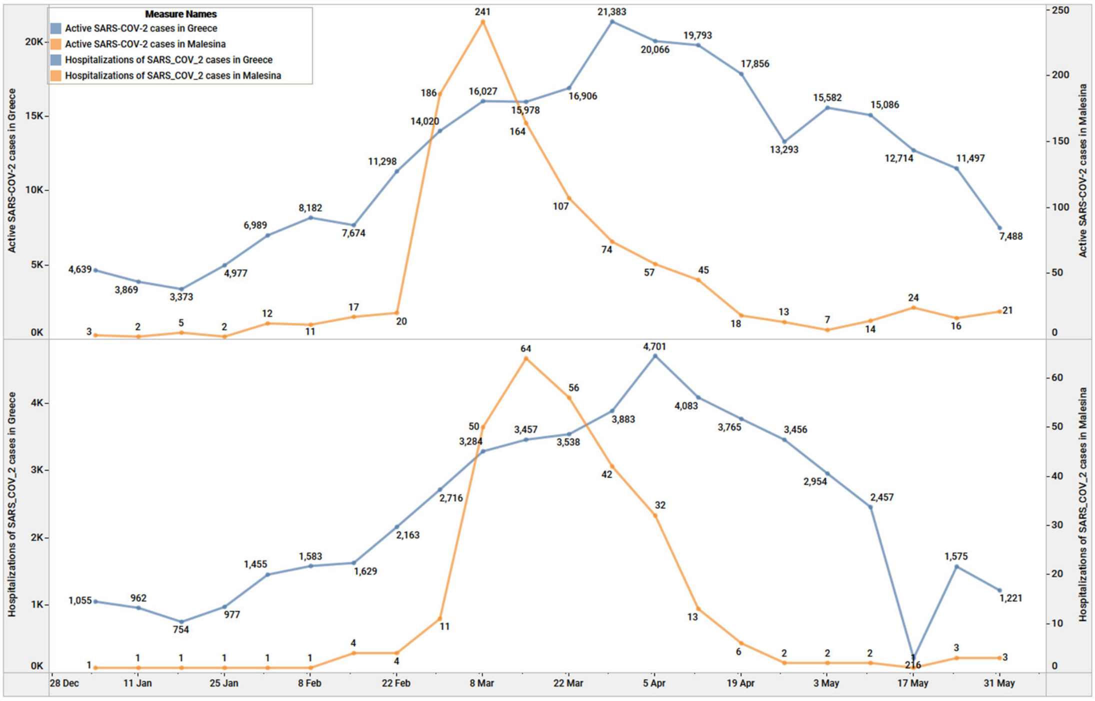

Figure S1. Comparisons of the active cases (upper graph) and hospitalizations between Malesina and the overall numbers for Greece (October 2020—May 2021).
